# Socio-demographic factors and neighbourhood social cohesion influence adults’ willingness to grant children greater independent mobility: A cross-sectional study

**DOI:** 10.1186/s12889-015-2053-2

**Published:** 2015-07-22

**Authors:** Stephanie Schoeppe, Mitch J. Duncan, Hannah M. Badland, Stephanie Alley, Susan Williams, Amanda L. Rebar, Corneel Vandelanotte

**Affiliations:** Physical Activity Research Group, School of Human, Health and Social Sciences, Central Queensland University, Building 18, Bruce Highway, Rockhampton, QLD 4702 Australia; Priority Research Centre for Physical Activity and Nutrition, Advanced Technology Centre, School of Medicine and Public Health, The University of Newcastle, ATC 315 University Drive, Callaghan, NSW 2308 Australia; McCaughey VicHealth Community Wellbeing Unit, The University of Melbourne, 207 Bouverie Street, Carlton, VIC 3010 Australia; School of Medical and Applied Sciences, Central Queensland University, Building 6, Bruce Highway, Rockhampton, QLD 4702 Australia

**Keywords:** Parent, Community cohesion, Unsupervised, Movement, Young people, Survey

## Abstract

**Background:**

In developed countries, children’s independent mobility levels are low. Built environmental factors and parental safety concerns are well-known to predict the level of independent mobility adults grant to children. In contrast, the influence of adults’ socio-demographic characteristics and neighbourhood social cohesion on children’s independent mobility is largely unexplored. This study investigated the influence of adults’ socio-demographic factors and neighbourhood social cohesion on distances they would permit children for independent travel and outdoor play.

**Methods:**

In 2013, a random sample of 1293 Australian adults (mean age: 56.1 years, 52 % male, 81 % parents) participated in the Queensland Social Survey (QSS) via computer-assisted telephone interview. Socio-demographic factors measured included age, sex, parental status, education and area-level socio-economic disadvantage. Perceived neighbourhood social cohesion was assessed using a standardised scale. Adults reported the distances children aged 8–12 years should be allowed to walk/cycle to places, and play outdoors without adults. Responses were categorised into ‘within sight’, < 0.5 kilometres (km) , 0.5-1 km and >1 km. Ordinal logistic regression was used to assess associations of socio-demographic factors and neighbourhood social cohesion with distances adults would permit for children’s independent travel and outdoor play.

**Results:**

Parents and adults with lower education were less likely to permit greater distances for children’s independent travel (OR = 0.57 and OR = 0.59, respectively). Women, parents and adults with lower education were less likely to grant children greater distances for independent outdoor play (OR = 0.61, OR = 0.50 and OR = 0.60, respectively). In contrast, adults with higher perceptions of neighbourhood social cohesion were more likely to permit children greater distances for independent travel (OR = 1.05)and outdoor play (OR = 1.05). Adult age and area-level socio-economic disadvantage were not associated with distances adults would permit for independent travel and outdoor play.

**Conclusions:**

Women, parents (particularly those of younger children), adults with lower education and those who perceived neighbourhood social cohesion as being lower were less willing to let children independently travel further away from home. Interventions to increase children’s independent mobility may be more effective if targeted to these groups. In addition, increasing neighbourhood social cohesion may help increase adults’ willingness to grant children greater independent mobility.

## Background

Independent mobility (i.e., unsupervised travel and outdoor play) provides children with many opportunities to increase physical activity [1–3]. This can help children achieve the recommended 60 minutes of moderate-to-vigorous physical activity a day [4, 5]. Independent travel and outdoor play promote children’s healthy development such as bone health, motor skills, physical fitness and healthy weight [6–9]. In addition, independent mobility provides children with psychosocial, cognitive and developmental benefits in the form of social interactions with peers, spatial and traffic safety skills for navigating in public spaces, and decision-making maturity [10–12].

Compared with previous generations, children are being granted significantly less freedom to move independently in public spaces [13]. For example, Australian data showed that between 1971 and 2003, the proportion of children who walked to school declined from 58 % to 35 %, whilst the proportion of children who were driven to school by car increased from 23 % to 67 % [14]. Similar declines in independent travel to school have been observed in other developed countries such as England, Germany, Finland, Norway and Denmark [15, 16]. A key marker of independent mobility is the distances children are allowed to travel independently in public spaces. Nowadays, the distances most Australian children travel independently tend to be short. For example, Veitch et al. [17] reported that 32 % of children aged 8–12 years independently walk or cycle < 0.1 km from home, 32 % travel 0.15-0.999 km and 36 % travel > 1 km from home. Moreover, the most frequently (74 %) used space for children’s outdoor play has been found to be the yard at home [18]. The reasons for low independent mobility levels in today’s children are manifold. Built environmental factors such as high traffic volume, lack of cycling and footpaths and greater distances to school and leisure-time destinations [19, 20] play a role, as do social environmental factors such as parental concerns about road safety, neighbourhood crime, bullying and stranger danger [21]. The influence of built environmental factors and neighbourhood safety concerns on adults willingness to grant children independent mobility have been widely investigated [19, 22, 23].

In contrast, the socio-demographic factors (adult age, sex, parental status, education, and level of socio-economic disadvantage) which might influence adults’ willingness to grant children independent mobility have received little attention in the literature [24]. Previous studies [19, 25–29] have mainly focused on child-related socio-demographics and consistently found that child age and sex determines parents’ willingness to grant children independent mobility. Adults including parents, grandparents, relatives, teachers and other child caregivers are considered gatekeepers to children’s independent travel and outdoor play, as they usually permit or restrict these behaviours [30]. Hence, adult-related socio-demographics may also be important predictors of children’s independent mobility in the neighbourhood.

Neighbourhood social cohesion is another potential predictor of children’s independent mobility which has received little attention [31]. Social cohesion describes the level of connectedness or solidarity among groups in a society [32]. For example, parental social networking and collective activity among families and residents may assure parents that the neighbourhood is a safe place for children’s outdoor play, or walking and cycling to school [33]. Neighbourhood places such as local streets, shops, cafés and recreational facilities have been described as being important ‘third places’ of social interaction after the home (first) and workplaces (second) [34]; and it is in these ‘third places’ where neighbourhood social cohesion develops [32]. It may be that high perceptions of neighbourhood social cohesion alleviate parental concerns about neighbourhood safety (stranger danger, crime, bullying), and thereby, increase their willingness to grant children greater independent mobility [35, 36]. However, few studies have examined the importance of neighbourhood social cohesion for children’s independent mobility [31, 37]. This study aimed to address these research gaps by investigating the influence adults’ socio-demographic factors and neighbourhood social cohesion have on the distances they would permit children to independently travel and play outdoors. This information can inform the development and targeting of public health interventions to promote independent walking, cycling and outdoor play of children.

## Methods

### Study population

Between July and August 2013, a random sample of 1293 Australian adults participated in the Queensland Social Survey (QSS) via computer-assisted telephone interview. The QSS is an omnibus survey of households in the state of Queensland, Australia administered by the Population Research Laboratory at Central Queensland University. Questions on socio-demographic factors, neighbourhood social cohesion and distances adults would permit for children’s independent travel and outdoor play were collected. Participants provided informed consent and the Human Ethics Committee at Central Queensland University approved the study.

#### Measures

##### Socio-demographic factors

Socio-demographic factors were measured including adult age, sex, parental status (parent of children 0–12 years, parent of children ≥ 13 years, non-parent), level of education (≤12 years, 13–14 years, ≥ 15 years) and post code of residence. Post codes were linked to the socio-economic index for areas (SEIFA) developed by the Australian Bureau of Statistics. This was used to provide an indicator of area-level socio-economic disadvantage. The SEIFA ranks community areas in Australia according to relative socio-economic disadvantage using census data on education, employment, occupation, housing and English proficiency [38]. SEIFA decile scores (ranging from 1 = lowest disadvantage to 10 = highest disadvantage) were grouped into low (1, 2 and 3 decile), medium (4, 5, 6 and 7 decile) and high (8, 9 and 10 decile) area-level socio-economic disadvantage.

##### Neighbourhood social cohesion

Perceived neighbourhood social cohesion was assessed through a five item scale [39, 40]. Adults rated their agreement with five statements about their neighbourhood on a 5-point Likert scale (1 = strongly disagree to 5 = strongly agree): ‘People in the neighbourhood are willing to help each other ‘The neighbourhood is a tight community ‘The people in the neighbourhood can be trusted ‘In general, the people in the neighbourhood get along well and ‘People in the neighbourhood share the same norms and values’. A sum score of ‘neighbourhood social cohesion’ was computed, ranging between 5–25; higher scores represent greater neighbourhood social cohesion. In this sample, the internal consistency of the scale, as measured by Cronbach’s alpha, was 0.81.

##### Distances adults would permit for children’s independent travel and outdoor play

Distances adults would permit for children’s independent active travel and outdoor play were assessed by two items. While not identical, this measure of independent mobility is comparable to those used previous studies [17, 18, 20]. Adults were asked ‘How far away from home should children aged 8–12 years be allowed to walk and cycle to places without an adult?’ Example places included schools, shops, friend's houses, sport and recreation centres. Adults were also asked ‘How far away from home should children aged 8–12 years be allowed to play in outdoor areas without an adult?’ Example outdoor areas included yards, streets, bush areas, open fields, parks and playgrounds. Response options for both questions were ‘within sight < 0.5 km (m), 0.5-1 kilometres (km), 1–2 km, 2–3 km, 3–5 km and > 5 km; responses were collapsed into the categories ‘within sight < 0.5 km, 0.5-1 km, and >1 km. The rationale for focusing on children aged 8–12 years was that children’s independent mobility usually increases during this age period when parents notice increasing physical and cognitive capabilities in their children [21, 25].

#### Statistical analyses

Ordinal logistic regression was used to assess associations of socio-demographic factors and neighbourhood social cohesion with the distances adults would permit for children’s independent travel and outdoor play. Proportional odds ratios (ORs), confidence intervals (CIs) and *p*-values were used as indicators of effect size and statistical significance of the adjusted associations between adult age, sex, parental status, level of education, area-level socio-economic disadvantage and neighbourhood social cohesion (predictor variables), and the odds of adults permitting greater distances for children’s independent travel and outdoor play. Variance inflation factors, R^2^-square values and parameter estimates were inspected to ensure there was no multicollinearity amongst predictor variables. The ordered logit models were run separately for the outcome variables ‘distances adults would permit for children’s independent travel’ (*N* = 1056) and ‘distances adults would permit for children’s independent outdoor play’ (*N* = 1051). Participants with missing data across variables were excluded from analyses (age: *N* = 9; education: *N* = 13; parental status: *N* = 5; area-level socio-economic disadvantage: 15; neighbourhood social cohesion: *N* = 147; independent travel: *N* = 78; independent outdoor play: *N* = 80). Chi-square and independent t-tests were performed to assess differences in variables between included and excluded participants. Analyses were performed in IBM SPSS Statistics (version 22.0) with significance levels set at *p* < 0.05.

## Results

Descriptive statistics for the socio-demographic variables, neighbourhood social cohesion and the distances adults would permit for children’s independent travel and outdoor play are presented in Table 1. The mean age was 56.1 (SD = 15.3) years, 52 % were male, 81 % were parents, 41 % had ≥ 15 years of education, and 34 % lived in residential areas with high socio-economic disadvantage. The mean sum score for level of neighbourhood social cohesion was 18.3 (SD = 3.4, range 5–25). Over one third of adults (36 %) reported that they would restrict 8–12 year old children’s independent travel to places that are within sight; 26 % of adults would permit independent travel to places < 0.5 km from the home, 18 % would allow distances of 0.5-1 km and 20 % of adults would permit > 1 km. Similarly, nearly half of adults (46 %) reported that they would restrict 8–12 year old children’s independent outdoor play to areas that are within sight; 28 % of adults would permit independent outdoor play in areas < 0.5 km from the home, 14 % would allow distances of 0.5-1 km and 12 % of adults would permit > 1 km. There were no significant differences in sex, parental status, area-level socio-economic disadvantage, neighbourhood social cohesion score and distances adults would permit for children’s independent travel and outdoor play between participants included and excluded from the analyses. Compared to included participants, excluded participants were older (56.1 vs 61.1 years vs; *p* < 0.001) and had lower levels of education (≤12 years: 44 % vs 55 %; 13–14 years: 15 % vs 8 %; ≥ 15 years: 41 % vs 37 %; *p* = 0.028).

**Table 1:** Descriptive statistics (*N* = 1293)

	All	Male	Female
Sex^a^	100.0	51.9	48.1
Age^b^	56.1 (15.3)	55.9 (15.7)	56.2 (14.8)
Parental status^a^			
Parent (children 0–12 years)	22.7	24.0	21.4
Parent (children ≥ 13 years)	57.8	55.1	60.7
Non-parent	19.5	20.9	18.0
Education^a^			
≤12 years	45.0	39.3	51.1
13-14 years	14.3	15.0	13.6
≥15 years	40.7	45.8	35.3
Neighbourhood social cohesion^b^	18.3 (3.4)	18.24 (3.3)	18.32 (3.4)
Area-level socio-economic disadvantage^a^			
Low	22.1	23.8	20.3
Medium	43.7	42.2	45.2
High	34.2	33.9	34.5
Distances adults would permit for children’s independent travel^a^			
Within sight	36.0	33.8	38.4
<0.5 km	26.2	23.9	28.6
0.5-1 km	18.3	19.2	17.3
>1 km	19.6	23.1	15.8
Distances adults would permit for children’s independent outdoor play^a^			
Within sight	45.6	39.6	52.1
<0.5 km	28.4	27.7	29.2
0.5-1 km	14.0	17.1	10.7
>1 km	12.0	15.5	8.1

^a^Percentage

^b^Mean (SD)

The results of the ordinal regression analyses examining associations between socio-demographic factors and neighbourhood social cohesion, and the odds of adults permitting greater distances for children’s independent travel and outdoor play are presented in Table 2. Adult age and area-level socio-economic disadvantage were not associated with distances adults would permit for children’s independent travel and outdoor play. However, significant associations were observed in relation to sex, parental status, education and neighbourhood social cohesion; these are presented below.

**Table 2:** Socio-demographic factors and neighbourhood social cohesion, and the odds of adults permitting greater distances for children’s independent travel and outdoor play

	Independent travel^a^		Independent outdoor play^b^	
	Odds ratio (95 % CI)	*P*	Odds ratio (95 % CI)	*P*
Age	1.00 (0.99-1.01)	0.498	1.00 (0.99-1.00)	0.231
Sex				
Female	0.81 (0.65-1.02)	0.068	**0.61 (0.48-0.76)**	**0.000**
Male	1.0		1.0	
Parental status				
Parent (children 0–12 years)	**0.57 (0.40-0.81)**	**0.002**	**0.50 (0.35-0.72)**	**0.000**
Parent (children ≥ 13 years)	0.87 (0.66-1.17)	0.360	0.85 (0.63-1.14)	0.279
Non-parent	1.0		1.0	
Education				
≤12 years	**0.59 (0.46-0.76)**	**0.000**	**0.60 (0.46-0.77)**	**0.000**
13-14 years	0.87 (0.63-1.22)	0.427	0.84 (0.60-1.17)	0.303
≥15 years	1.0		1.0	
Area-level socio-economic disadvantage				
Low	0.86 (0.63-1.16)	0.319	0.90 (0.66-1.23)	0.496
Medium	0.85 (0.66-1.09)	0.193	0.97 (0.75-1.26)	0.840
High	1.0		1.0	
Neighbourhood social cohesion	**1.05 (1.01-1.08)**	**0.008**	**1.05 (1.02-1.09)**	**0.005**

Odds Ratios in bold are significant.

^a^ *N* = 1056

^b^ *N* = 1051

### Independent travel

Parental status, level of education and perceived neighbourhood social cohesion were significantly associated with distances adults would permit for children’s independent travel (Table 2). Parents of younger children (0–12 years) were less likely to permit children greater distances for independent travel (OR = 0.57, 95 % CI: 0.40-0.80) than parents of older children (≥13 years) and non-parents. Adults with lower education were less likely to grant children greater travel distances (OR = 0.59, 95 % CI: 0.46-0.76) than adults with higher education. Adults with higher perceptions of neighbourhood social cohesion were more likely to permit children greater distances for independent travel (OR = 1.05, 95 % CI: 1.01-1.08) than adults with lower perceptions of neighbourhood social cohesion. Further, women were also less likely to grant children greater travel distances (OR = 0.81, 95 % CI: 0.65-1.02) than men; however, this associations did not reach statistical significance (*p* = 0.068).

### Independent outdoor play

The distances adults would permit for children’s independent outdoor play differed significantly as a function of sex, parental status, level of education and perceived neighbourhood social cohesion (Table 2). Women (OR = 0.61, 95 % CI: 0.48-0.76), parents of younger children (0–12 years; OR = 0.50, 95 % CI: 0.35-0.72) and adults with lower education (OR = 0.60, 95 % CI: 0.46-0.77) were less likely to allow children greater distances for independent outdoor play than men, parents of older children (≥13 years), non-parents and adults with higher education. In contrast, adults with higher perceptions of neighbourhood social cohesion (OR = 1.05, 95 % CI: 1.02-1.09) were more likely to permit children greater distances for independent outdoor play than adults with lower perceptions of neighbourhood social cohesion.

## Discussion

This study investigated the influence of socio-demographic factors as well as neighbourhood social cohesion on the distances adults would permit for children’s independent travel and outdoor play. Overall, findings showed that distances adults would permit for children’s independent travel differed significantly as a function of parental status, education and neighbourhood social cohesion. Further, the distances adults would permit for children’s independent outdoor play differed significantly as a function of sex, parental status, education and neighbourhood social cohesion. In contrast, there was no impact of age or area-level socio-economic disadvantage on the distances adults would permit for independent travel or outdoor play.

Women were less likely to permit children greater distances for independent outdoor play than men. This outcome is in line with previous research which demonstrates that in general women are more cautious than men [41, 42] and they spend more time supervising children than men [43]; hence, they may be more vigilant to potential safety hazards associated with independent travel and outdoor play. In contrast, men tend to be more comfortable with letting children engage in outdoor activities that contain some risk and independence [42]. It is worth noting that women were also less likely to grant children greater distances for independent travel; however, this relationship did not reach statistical significance. Possibly, women consider independent walking and cycling on pavements less hazardous than independent outdoor play on streets, bush areas, open fields and playgrounds.

Parents of younger children were less likely to permit children greater distances for independent travel and outdoor play than parents of older children and non-parents Possibly, the close parent–child bond (e.g., ‘maternal instinct’) and parents’ ‘trained eye’ to hazards in young children’s environment leads parents to feel a greater need to protect their offspring from potential dangers surrounding outdoor activities. In contrast, parents of older children (who may already be adults) and non-parents may perceive risks and safety issues as less serious. Furthermore, social and cultural norms about good parenting and child care have been shown to influence adult restrictions to children’s independent mobility [44]. For example, in many societies close supervision of children is recognised as good parenting, whereas letting children roam freely signals poor parenting [23, 45].

The finding that adult age had no impact on the distances adults would permit for children’s independent travel and outdoor play was somewhat surprising. Given that in previous generations children were granted more independent mobility [13–15], it was anticipated that older adults would be more likely to permit greater distances for independent travel and outdoor play than younger adults. However, this was not the case, and the descriptive results showed that over two thirds of adults in this sample would restrict children’s independent travel and outdoor play to less than 500 m from the home. Possibly, adults from all generations consider neighbourhoods less safe nowadays [46], which may be driven by increases in traffic volume/speed [47], as well as concerns about crime, bullying and stranger danger [23, 48].

Adults with lower education were less likely to permit children greater distances for independent travel and outdoor play than adults with higher education. Comparative studies are lacking, hence, it is hard to explain these findings. Panter et al. [4] found contrasting results showing that children of parents with lower education were more likely to take up active travel; however, their study did not investigate whether children travelled independently, and the distances they were travelling. One explanation for our finding could be that, compared to adults with higher education, adults with lower education live in neighbourhoods that are perceived as less safe for independent travel and outdoor play. Although contrary to this, our study showed that area-level socio-economic disadvantage (which includes education) did not influence adults’ willingness to grant children greater independent mobility. Such influence was plausible given that area-level socio-economic disadvantage is considered a predictor of neighbourhood crime rates [49]. Another explanation may be that adults with lower education may not recognise the importance of independent travel and outdoor play for children’s healthy development, such as good motor skills and healthy weight from being physically active [50], social integration with peers and maturity for navigating in public spaces [10, 11] causing them to restrict these behaviours to a narrow geographic range.

Adults with higher perceptions of neighbourhood social cohesion (characterised by friendliness, helpfulness, trust, shared norms and values) were more likely to permit children greater distances for independent travel and outdoor play than adults with lower perceptions of neighbourhood social cohesion. This is consistent with the results from similar studies [51–53] showing that a stronger sense of community and the presence of more social relations among parents was associated with greater independent mobility in children. Higher neighbourhood social cohesion, irrespective of area-level disadvantage, may reflect adults’ consideration of their neighbourhood as a safe place for walking, cycling and outdoor play for children.

This was the first study to investigate the influence of socio-demographic factors on adults’ willingness to grant children greater independent mobility. Another novel aspect in this study was the examination of area-level socio-economic disadvantage and neighbourhood social cohesion as a predictor of child independent mobility which have rarely been examined before. Other methodological strengths of this study include the use of a large random population sample, standardised measures of neighbourhood social cohesion, as well as the focus on independent travel and outdoor play. Both constitute independent mobility but have rarely been examined simultaneously in previous studies [20, 54]. This study also had limitations. First, most participants were older (mean age 56 years); a sample with more equal proportions of younger and older adults may have been more suitable for examining associations by age. Second, the QSS targeted Queensland adults; therefore, findings may not be generalisable to the general Australian population. Third, we assessed adult attitudes on what distances are appropriate for children aged 8–12 years to travel independently. From this we cannot infer distances children are actually allowed to travel independently, and whether distances children are allowed to travel differ if travelled alone or with other children. However, adult attitudes on acceptable distances for children’s independent travel and outdoor play presented in this study reveal common attitudes and social norms in relation to children’s independent mobility, and these are likely to influence children’s independent mobility levels. We measured adult perceptions of neighbourhood social cohesion; these are subjective and may not reflect actual neighbourhood social cohesion. Although, it is likely that adults’ perception of neighbourhood social cohesion will determine their judgment of the neighbourhood as a safe place for independent travel and outdoor play.

Given that adults usually decide what distances children are allowed to travel independently, the influence of adults’ socio-economic characteristics (e.g., employment status, marital status, household income) on their willingness to grant more independent mobility to children is worth exploring further in future studies. Such information may help target particular parent populations in interventions to promote active travel and outdoor play in children. Interventions in this area may incorporate strategies to challenge social norms and educate parents and their children that the health and social benefits of independent mobility may outweigh the inflated risks publicised in the media [23]. It may also be worth exploring whether children’s past behaviours and temperaments influence parents’ willingness to grant independent mobility.

Future studies should test effectiveness of strategies to promote neighbourhood social cohesion (e.g., increasing neighbour relations and mutual trust, community activities and social networks) in increasing parents’ willingness to grant children greater independent mobility. However, such strategies may be more effective if supported by environmental interventions such as the provision of foot and cycling paths, and traffic calming measures.

## Conclusions

Parents of younger children and adults with lower education were less inclined to let children travel and play outdoors further away from home. Women and parents of younger children were also less willing to let children play outdoors further away from home. In contrast, adults with higher perceptions of neighbourhood social cohesion were more willing to permit children greater distances for independent travel and outdoor play. These observations suggest that interventions to increase children’s independent mobility should particularly target women, parents (particularly those of younger children) and adults with lower education. A possible intervention strategy may be to educate mothers on the health and social benefits of children’s independent mobility. Moreover, the promotion of neighbourhood social cohesion such as by increasing neighbour relations and social networks may help assure parents that the neighbourhood is safe for children’s unsupervised travel and outdoor play.

## Abbreviations

QSS: Queensland Social Survey
SEIFA: Socio-economic index for areas
